# The Anti-Angiogenic Effect of *Cynara cardunculus* L. subsp. *cardunculus* Waste Product

**DOI:** 10.3390/foods14152656

**Published:** 2025-07-29

**Authors:** Anna Cacciola, Valeria D’Angelo, Federica De Gaetano, Antonella Fais, Maria Paola Germanò, Valentina Masala, Stefania Olla, Venerando Pistarà, Rosanna Stancanelli, Carlo Ignazio Giovanni Tuberoso, Cinzia Anna Ventura

**Affiliations:** 1 Department of Chemical, Biological, Pharmaceutical and Environmental Sciences, University of Messina, Viale F. Stagno d’Alcontres 31, 98166 Messina, Italy; anna.cacciola@unime.it (A.C.); valeria.dangelo@unime.it (V.D.); federica.degaetano@unime.it (F.D.G.); rosanna.stancanelli@unime.it (R.S.); cinziaanna.ventura@unime.it (C.A.V.); 2 Department of Life and Environmental Sciences, University of Cagliari, SS 554-bivio per Sestu, 09042 Monserrato, Italy; fais@unica.it (A.F.); valentina.masala2@unica.it (V.M.); 3 Institute for Genetic and Biomedical Research (IRGB), The National Research Council (CNR), 09042 Monserrato, Italy; stefania.olla@irgb.cnr.it; 4 Department of Pharmaceutical and Health Sciences, University of Catania, Viale Andrea Doria 6, 95125 Catania, Italy; vpistara@unict.it

**Keywords:** wild cardoon, collagenase, angiogenesis, chick chorioallantoic membrane, zebrafish, hydroxypropyl-β-cyclodextrin

## Abstract

*Cynara cardunculus* L. subsp. *cardunculus* (*Cynara cardunculus* L. var. *sylvestris* (Lam.) Fiori), the wild cardoon, is known for its culinary applications and potential health benefits. Due to this, and given the growing interest in circular economies, deepening our under-standing of the effects of wild cardoon leaf waste on angiogenesis and collagenase activity represents a valuable opportunity to valorise agricultural byproducts as health-promoting ingredients. In this study, the waste product of wild cardoon leaves was extracted to examine its chemical composition and biological activities. Analytical techniques identified several bioactive compounds, including flavonoids, hydroxycinnamic acids such as dicaffeoyl-succinoylquinic acids, and luteolin-7-*O*-rutinoside. In vivo tests in zebrafish embryos and the chick chorioallantoic membrane demonstrated dose-dependent antiangiogenic effects, particularly enhanced by the complexation with hydroxypropyl-β-cyclodextrin (HP-β-CD). Considering the link between angiogenesis and collagenase, the potential effects of the extract on collagenase activity was investigated. The extract alone inhibited collagenase with an IC_50_ value comparable to that of the standard inhibitor while its complexed form exhibited a 4.5-fold greater inhibitory activity. A molecular docking study examined the interaction between the main compounds and collagenase. In conclusion, wild cardoon leaves can represent a valuable source of bioactive compounds. This study demonstrated that the complexation of the extract with cyclodextrin determines an increase in its biological activity.

## 1. Introduction

The agri-food sector generates a considerable amount of by-products every year, which are often wasted, resulting in the loss of valuable resources. By-products valorisation is fundamental for the effective application of the circular economy and global sustainability [1]. Since numerous by-products from the agricultural or food industry contain bioactive molecules with a high number of properties useful in the prevention of many important diseases, they have recently garnered considerable interest for their reuse and the production of bioactive compounds, as well as for use as natural additives [2,3].

The large amounts of waste produced by packing houses and the food-processing industry can reach ∼60% of the harvested vegetable, as in the case of the industrial manipulation of artichokes and cardoons [4]. For this reason, the artichoke and cardoon supply chain has attracted increasing attention for the valorisation of its by-products. From an agronomic perspective, *C. cardunculus* species—especially in their cultivated and wild forms—generate considerable amounts of leaf biomass, much of which is discarded during industrial processing [5]. The sustainable valorisation of these by-products is gaining increasing attention considering the principles of the circular bioeconomy, which promotes the conversion of agricultural residues into high-value resources. In this context, *Cynara cardunculus*, belonging to the Asteraceae family, comprises three taxa: globe artichoke (var. *scolymus*), cultivated cardoon (var. *altilis*), and the wild cardoon (subsp. *cardunculus*). The wild perennial cardoon, *C. cardunculus* L. subsp. *cardunculus,* previously known as var. *sylvestris*, is a Mediterranean plant that thrives in warm, humid climates, which belongs to a multifunctional taxon recognised for its phytochemical richness, nutraceutical potential, and traditional medicinal uses [6]. Among these, the significance of wild cardoon leaf phenolic composition for human health, plant defence, and possible nutraceutical uses has attracted growing scientific attention. The medicinal potential of this wild taxon is greatly enhanced by phenolic compounds, a varied class of secondary metabolites. These compounds are well-known for their antibacterial, anti-inflammatory, and antioxidant qualities [7,8]. *C. cardunculus* L. subsp. *cardunculus*, a wild relative of the farmed cardoon and globe artichoke, has a distinct and frequently more chemically varied phenolic profile, which is probably affected by its ecological habitat and evolutionary forces [9]. These molecules determine the value of the plant as a source of bioactive compounds in pharmaceutical and functional food formulations, in addition to playing crucial roles in the plant’s physiological responses [8]. These metabolites can now be precisely identified and quantified due to recent developments in phytochemical analysis, particularly high-performance liquid chromatography coupled with mass spectrometry (HPLC-MS), making it easier to analyse them in detail [9].

Concerning its biological activity, Acquaviva et al. showed that the wild cardoon leaf extract helps reduce transient or mild hepatic oxidative stress [10]. In addition, wild thistle leaves have been proven to exhibit antibacterial properties [6]. Supporting its nutraceutical potential, dietary supplementation with wild cardoon leaf extract in rats showed a hypolipidemic effect [11]. Several biological activities are attributed to all varieties of *C. cardunculus*, among which are those shown by the methanolic extract of leaves. These have shown both antioxidant activity and the inhibition of proliferation and migration of tumour cells, as well as angiogenesis in vivo [12]. The process of angiogenesis is essential for growth, development, and wound healing and is implicated in several pathological conditions. This mechanism consists of the formation of new vessels from existing ones [13]. Moreover, collagenase is involved in the angiogenic process and plays a role in collagen remodelling [14]. Therefore, inhibiting collagenase activity may improve therapeutic outcomes in managing angiogenesis-related diseases [15]. Velez et al. [12] have highlighted that the methanolic extract of cultivated cardoon exhibits anti-angiogenesis properties. Despite this, to the best of our knowledge, no comprehensive studies have investigated the potential of wild cardoon leaf extracts to modulate collagenase activity and angiogenesis. Another critical limitation in the consumption and therapeutic application of bioactive molecules derived from artichoke and cardoon extracts lies in their unfavourable physical-chemical properties, which significantly influences their ability to cross the viable membranes, resulting in low and erratic bioavailability. For this reason, different delivery systems were studied aimed at improving the biopharmaceutical properties of natural products, and among them, cyclodextrins (CDs) have received great attention. They are cyclic oligosaccharides able to form inclusion and non-inclusion complexes with both synthetic molecules [16,17] or bioactive compounds derived from natural sources [18,19,20]. Specifically, hydroxypropyl-β-cyclodextrin (HP-β-CD) has shown superior capacity to encapsulate polyphenols from vegetal extracts, resulting in improved oral bioavailability, enhanced antioxidant activity, and sustained biological effects, permitting their application in medicine, cosmetics, and the food industry [21,22]. Lipophilic molecules can accommodate into the cavity of the macrocycle and their physical-chemical properties were improved, such as solubility and stability. The complex quickly dissolves in the physiological environment, improving the availability of the bioactive molecules at the surface of the biological barrier. As a result, the concentration gradient through the membrane was improved, and absorption was enhanced. Furthermore, it is now proven that CDs can act as penetration enhancer agents by interacting with the viable membranes and extracting their components (cholesterol and lipids), thus improving permeability to both lipophilic and hydrophilic drugs [23].

To support this, there are several studies in the literature that testified the effective use of CDs for the encapsulation and delivery of natural compounds [24,25]. Zhong et al. [26] investigated the activity of inclusion complexes between tea polyphenols and HP-β-CD on myofibrillar protein (MP) extracted from lamb tripe and reported a significant enhancement in the antioxidant activity and bioavailability of the inclusion complexes (5–105 µmol/g). This improvement was attributed to a reduction in carbonyl content, hydrophobicity, and the protein aggregation of MP, as well as an increase in sulfhydryl group content. Furthermore, Li et al. [27] demonstrated that the formation of inclusion complexes between curcumin and HP-β-CD doubles the plasma concentration of the molecule, thereby enhancing its bioavailability compared to the free form. In addition, these complexes maintain more stable plasma levels over time, thus prolonging the beneficial effects of curcumin.

Although the health-promoting properties of the wild cardoon are well-documented, its leaf residues remain underexplored for potential use in the functional food sector, with most research focused on pharmaceutical applications. Addressing this gap could support the development of new nutraceutical ingredients and food-based strategies aimed at cardiovascular and metabolic health. In this context, the present study aims to chemically characterise by ^1^H NMR, LC-MS/MS and LC-PDA the extract obtained from the wild cardoon leaf by-products and to evaluate its collagenase inhibitory activity and antiangiogenic potential. The antiangiogenic effects were investigated through in vivo assays using the chick chorioallantoic membrane (CAM) and zebrafish (*Danio rerio*) embryo models. Additionally, the extract was complexed with hydroxypropyl-β-cyclodextrin (HP-β-CD) to assess the impact of this formulation strategy on biological activity. Finally, molecular docking was conducted to explore the interactions between the target phenolic constituents and collagenase.

## 2. Materials and Methods

### 2.1. Plant Material and Extraction Method

*Cynara cardunculus* L. subsp. *cardunculus* leaf by-products were collected in March 2024 in Serrenti (South Sardinia, Italy). The specimens were identified by Prof. Maria Paola Germanò (University of Messina, Italy), and a voucher sample (number DISVA.ALI.01.2024) was deposited at the Department of Life and Environmental Sciences of the University of Cagliari (Italy). The leaves were carefully cleaned and the hardest parts were removed and prepared for the extraction. From the sample of about 5 kg, extracts were obtained with EtOH:H_2_O 80:20% *v*/*v* according to the following procedure: 200 g of fresh leaves were extracted with 500 mL of solvent mixture, initially using an immersion blender and subsequently completing the extraction with an Ultra Turrax IKA T18 digital (IKA-Werke GmbH & Co. KG, Staufen, Germany). The obtained extract (SyEt80) was coarsely filtered and centrifuged for 15 min at 4000 rpm at 10 °C. The supernatant was concentrated under vacuum to remove the ethanol using a Rotavapor^®^ R-210 (BUCHI, Cornaredo, Italy) with a heated bath set at t < 40 °C. The extract thus obtained was centrifuged for 15 min at 4000 rpm at 10 °C, frozen at −20 °C, and finally freeze-dried using a Lio5P freeze dryer (5Pascal, Trezzano sul Naviglio, Italy).

### 2.2. ^1^H NMR Analysis

Nuclear Magnetic Resonance (NMR) spectra were recorded on a Varian Unity Inova 500 MHz (11.75 T) at 300 K using a 5 mm multinuclear direct detection probe. All spectra were taken in D_2_O as solvent. The chemical shifts are indicated as δ ppm values, and the residual water peak (4.79 ppm) was used as the internal reference.

### 2.3. Identification and Quantification of Polyphenolic Compounds

The SyEt80 was evaluated both qualitatively and quantitatively by (HR) LC-ESI-QToF-MS/MS and LC-PDA using the methodology described by De Luca et al. [28] and Masala et al. [29]. The studies were conducted using an electrospray ionisation (ESI) source that was set up to function in both positive and negative ion modes. In summary, the analytical set up consisted of an advanced ion mobility QToF LC/MS system including a 1290 Infinity II UPLC and a 6560 IM-QToF (Agilent Technologies Inc., Palo Alto, CA, USA). The ESI/QToF MS data were then assessed using the MassHunter Workstation Qualitative Analysis program, version 10.0 (Agilent Technologies). For the tentative identification of the metabolites and to predict fragmentation and molecular formulae, the MassHunter METLIN metabolite PCDLdatabase B.08.00 (Agilent Technologies), comparison between the experimental MS/MS spectra and spectra from a publicly available mass spectral data repository (e.g., Sirius^®^ software v. 5.5.5 [30,31] and MZmine^®^ v. 4.3 [32]), and fragmentation patterns that have been published in the literature and other natural products databases (KNApSAcK^®^, PubChem^®^, Coconut^®^) were used. Phenolic substances were quali-quantitatively analysed using a 1260 Infinity II HPLC system and an Agilent Technologies G4212B photodiode array detector. The separation was obtained with a Kinetex EVO C18 column (150 × 4.60 mm, 2.6 μm, Phenomenex, Castel Maggiore, BO, Italy) using 0.22 M phosphoric acid (solvent A) and acetonitrile (solvent B) as the mobile phase properly mixed in gradient elution at a constant flow rate of 0.8 mL/min. After decreasing solvent A from 100% to 80% in 20 min, 70% in 35 min, and 0% in 45 min, the gradient was returned to 100% solvent A and maintained for 5 min prior to the subsequent injection. The injection volume was 10 μL. The chromatograms and spectra were processed using Agilent Technologies’ OpenLab CDS software version 2.5. The calibration curves were built with the external standard method, using the least squares approach to correlate the peak area with the concentration, with R^2^ > 0.999 for all standards in the 0.2–10.0 mg/L range and data were expressed as mg/g dry extract residue (mg/g dr). The extracts were diluted 1:5 using 0.22 M phosphoric acid for the analysis. Before injection, the solutions were filtered using a 0.22 μm CA syringe filter.

### 2.4. In Silico Studies

#### 2.4.1. Protein and Ligand Preparation

In the Protein Data Bank (RCSB PDB) [33], the following two X-ray structures were selected: one corresponding to human fibroblast collagenase (PDB code: 1CGL; https://www.rcsb.org/structure/1CGL, accessed on 3 February 2025) and the other to collagenase H from *Clostridium histolyticum* (PDB code: 7ZOC; https://www.rcsb.org/structure/7ZOC, accessed on 3 February 2025). The structures were processed using the Protein Preparation Wizard tool (Schrödinger Release 2024-3) [34]. During this step, hydrogen atoms were added, bond orders were corrected, and formal charges were assigned. The appropriate ionisation state was determined at pH 7.4 using the PROPKA tool (https://www.ddl.unimi.it/vegaol/propka.htm, accessed on 3 February 2025) [35]. A subsequent energy minimisation was performed using the OPLS4 force field to resolve molecular overlaps and structural strains. The minimisation process was terminated when the root mean square deviation (RMSD) of the non-hydrogen atoms reached 0.3 Å. Seven compounds (epigallocatechin gallate with co-crystallised compounds used as a control and 1,5-dicaffeoyl-3-succinoylquinic acid, 1,5-dicaffeoyl-4-succinoyl quinic acid, chlorogenic acid, and luteolin-7-*O*-rutinoside) were retrieved from PubChem and prepared with LigPrep (Schrödinger Release 2024-3) using the OPLS4 force field and preserving the specified chirality.

#### 2.4.2. Molecular Docking

Molecular docking was performed using the Glide software (Schrödinger Release 2024-3 Commercial Version) in extra precision (XP) mode, and XP docking scores were used to rank ligand binding affinities [36,37]. Three poses per ligand were retained using a threshold of 0.50 kcal/mol, and the OPLS_2005 [38] force field was employed. To create grids, the Receptor Grid Generator was utilised. The centre of the grid, with dimensions of 46 × 46 × 46 Å, was determined based on the centroid of the residues within the binding site.

### 2.5. Preparation of the Extract/HP-β-CD Complex

#### 2.5.1. Preparation of the of SyEt80/HP-β-CD Complex

The complex was prepared following the lyophilisation method. Briefly, the extract (30 mg) was dissolved in 10 mL of water containing 0.5% *w*/*v* HP-β-CD. The solution was kept under continuous magnetic stirring at 500 rpm for 30 min and subsequently subjected to lyophilisation for 72 h using a Christ Alpha 2-4 LSCbasic freeze-dryer (Martin Christ Gefriertrocknungsanlagen GmbH, Osterode am Harz, Germany) to obtain a powder formulation. The lyophilised product was stored in a desiccator at room temperature until further analysis.

#### 2.5.2. Thermogravimetric Analysis of the Complex

A Discovery SDT 650 (TA Instruments—Waters LLC, Milford, MA, USA) was used to carry out TGA on the complex, comparative to the free extract and HP-β-CD. The analyses were performed in the temperature range 25–800 °C and a heating rate of 20.0 °C/min. A nitrogen flow rate of 100.0 mL/min was used. Each sample was weighed (5–10 mg), poured onto a ceramic plate, and analysed in triplicate.

### 2.6. Collagenase Activity Assay

Collagenase derived from *Clostridium histolyticum* (Sigma-Aldrich, Merk Life Science S.r.l., Milan, Italy) at a concentration of 1 U/mL was prepared in a Tricine buffer containing 50 mM tricine, 10 mM calcium chloride, and 400 mM sodium chloride, with a pH adjusted to 7.5. This solution was subsequently incubated both as a free extract and as an HP-β-CD (0.5% *w*/*v*) inclusion complex at various concentrations for a duration of 15 min. Following the incubation, the synthetic substrate N-(3-[2-Furyl]-acryloyl)-Leu-Gly-Pro-Ala (FALGPA) was introduced to achieve a final concentration of 0.8 mM. The absorbance of the resulting mixture was then monitored at a wavelength of 340 nm. Epigallocatechin gallate was utilised as a positive control in this assay.

### 2.7. In Vivo Studies

#### 2.7.1. Chick Chorioallantoic Membrane (CAM) Assay

The antiangiogenic activity of SyEt80, both as free extract and as a HP-β-CD (0.5% *w*/*v*) inclusion complex, was evaluated using the chick chorioallantoic membrane (CAM) assay, following the protocol described by Cioni et al. [39], with minor modifications. Fertilised chicken eggs were treated with the extract or the cyclodextrin complex at concentrations of 25, 50, and 100 μg/egg. Retinoic acid (1.0 μg/egg) was used as a positive control. After 48 h post-treatment the CAMs were observed using a stereomicroscope (SMZ-171 Series, Motic, Xiamen, China) equipped with a digital camera (Moticam^®^ 5 plus). Angiogenesis was assessed by quantifying vascular branch points within a standardised area using AngioQuant (software version v1.33, 2005). Results were expressed as mean values ± standard deviation (SD).

#### 2.7.2. Zebrafish Embryo Assay

The in vivo antiangiogenic effects of SyEt80, both in its free form and formulated with HP-β-CD (0.5% *w*/*v*), were further investigated in zebrafish (*Danio rerio*) embryos following the methodology reported by Cioni et al. [39]. Initially, the dechorionated embryos were treated only with CD to ensure its tolerability. At the chosen dose of 0.5% (*w*/*v*) it showed no signs of mortality or developmental alterations. Subsequently, dechorionated embryos were treated with the extract, either in the free form or as an inclusion complex with HP-β-CD (0.5% *w*/*v*) at concentrations of 10, 20, and 40 μg/embryo for 48 h. These concentrations were selected based on preliminary experimental studies aimed at identifying a biologically active range with acceptable safety margins. Concentration above 40 μg/embryo were associated with signs of developmental toxicity, whereas lower concentrations (<10 μg/embryo) did not cause a significant antiangiogenic response, even in the presence of HP-β-CD. Therefore, the selected range (10–40 μg/embryo) represents an optimal balance between efficacy and tolerability. This selection is further supported by similar dose ranges employed in zebrafish models for testing polyphenol-rich extracts and angiogenesis modulators [39]. 2-Methoxyestradiol (2 μM) was used as the positive control. All experiments were conducted in compliance with the European Directive 2010/63/EU and the ethical guidelines described in the “National Institutes of Health Guide for Care and Use of Laboratory Animals”. The experiments conducted on zebrafish embryos up to 72 hpf did not require ethical approval.

#### 2.7.3. Evaluation of Endogenous Alkaline Phosphatase (EAP) Activity in Zebrafish Embryos

The evaluation of endogenous alkaline phosphatase (EAP) activity in zebrafish embryos was performed according to the method described by De Leo et al. [40]. At 72 hpf, embryos underwent ethanol dehydration and were incubated with *p*-nitrophenyl phosphate (pNPP). The reaction was stopped with NaOH (2N), and absorbance was measured at 405 nm using a microplate spectrophotometer (AMR-100 T, ALLSHENG).

### 2.8. Statistical Analysis

Results were expressed as mean ± SD (standard deviation). Statistical analysis was performed using Student’s *t*-test using SigmaPlot 12.0 software to compare the means obtained in the different experiments, and results with * *p* < 0.05 or ** *p* < 0.01 were considered statistically significant.

## 3. Results and Discussion

### 3.1. Quali-Quantitative Determination of Bioactive Compounds in Wild Cardoon Leaf Extracts

**Figure 1:**
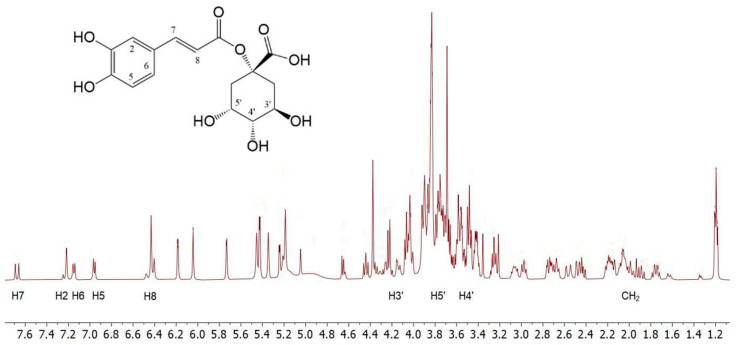
^1^H NMR spectrum portions of the extract; for simplicity, only the signals relating to caffeoylquinic acid have been indicated (the scale is in ppm).

To obtain preliminary information on the composition of *C. cardunculus* L. subsp. *cardunculus* extract, the ^1^H NMR analysis was conducted by dissolving the crude oily sample (2 μL) in 800 μL of D_2_O. The spectra showed complex mixtures that did not permit precise NMR characterisation of the extracts. Therefore, the structures of the main components were identified by comparing their ^1^H NMR data with those reported in the literature [41]. The ^1^H NMR spectrum (Figure 1) displays a pattern of signals similar to that of 1-caffeoylquinic acid. The caffeic acid moiety was confirmed by the protons of the 1,3,4-trisubstituted aromatic ring; H-6 and H-7 protons resonate at 7.16 and 7.15 ppm, respectively, as two doublets with typical aromatic proton *ortho* coupling constants (*J* = 8.0 Hz), while H-2 resonates at 7.22 ppm. The remaining two H-7 and H-8 unsaturated cinnamic protons appear at 7.68 and 6.42 ppm, respectively, and resonate as two doublets with a *trans* coupling constant (*J* = 16.0 Hz). In the region between 1.55–2.40 ppm and 3.8–4.25 ppm, a series of multiplets characteristic of the four methylene protons and the H-3′, H-4′, and H-5′ protons of a cyclohexanecarboxylic unit can be identified, which are associated with the quinic acid moiety.

Furthermore, in the same ^1^H NMR spectrum resonances for the protons of the two pyran rings of the rhamnose unit, along with those of the flavonoid unit, can be detected, indicating a structure of the luteolin 7-*O*-rutinoside type [41].

**Figure 2:**
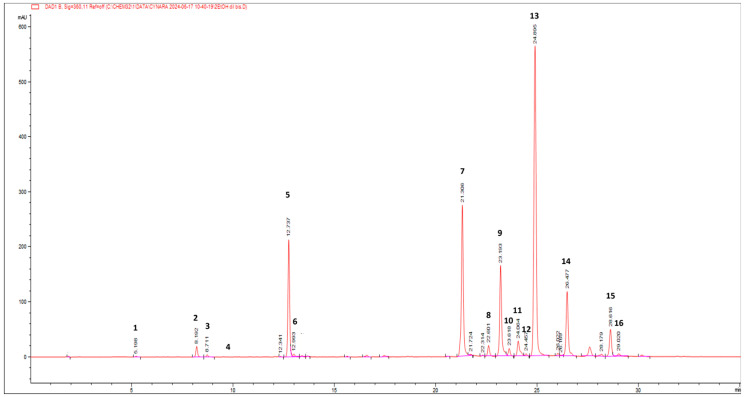
HPLC-PDA chromatogram at 360 nm of wild cardoon leaf extract (SyEt80). Chromatographic conditions are described in the text. Peak identifications are given in Table 1 and text.

Following this information, the leaf by-products’ extract was qualitatively analysed by (HR) LC-ESI-QTOF MS/MS in the negative and positive ion modes. The compounds were identified by comparing the experimental MS/MS spectra with the fragmentation patterns of both pure standard compounds and those reported in the literature and in the public repository of mass spectral data, as well as by comparing the *m/z* values with those described in the literature [29,30,31]. Additionally, compounds were identified according to their UV-Vis spectra and quantified by measuring their absorption at distinctive wavelengths (flavonols at 360 nm, hydroxycinnamic acids at 313 nm, hydroxybenzoic acids at 280 nm). Figure 2 reports the HPLC-PDA chromatogram at 360 nm of wild cardoon leaf extract (SyEt80), and peaks are identified according to the numeration reported in Table 1.

Table 1 reports the quantification of the target compounds by the LC-PDA method (amount expressed as mg/g dr). The identified phenolic compounds belong to the classes of hydroxycinnamic acids (mono caffeoyl quinic acids (peaks 2, 3, 5, and 6), dicaffeoyl-quinic acids (peaks 8, 9, 10, and 12), dicaffeoyl-succinoyl quinic acids (peaks 13 and 14), flavonoids (luteolin derivatives, peaks 7, 11, and 16), and hydroxybenzoic acids (protocatechuic acid hexoside (peak 1) and syringic acid hexoside (peak 4)). The most abundant class of compounds was represented by hydroxycinnamic acids, showing an average value of 75.5%, followed by flavonoids and hydroxybenzoic acids (24.0% and 0.5%, respectively). Concerning the most concentrated compounds, following the MS and UV-vis data, peak 13 at rt = 24.9 min was attributed to a dicaffeoyl-succinoyl quinic acid due to the specific [M − H]^−^ at *m/z* 615 and fragments at 515 (loss of a di-caffeoylquinic acid unit), 191 (loss of a quinic acid unit), and 179 (loss of a caffeic acid unit). By comparison with literature data on *C. cardunculus* subsp. *cardunculus* [42] and also on other varieties (var. *altilis* and var. *scolymus*) [43], peak 13 was tentatively attributed to 1,5-dicaffeoyl-3-succinoylquinic acid with an amount of 38.64 ± 0.21 mg/g dr. The second most concentrated compound was identified as luteolin 7-*O*-rutinoside (31.56 ± 0.38 mg/g dr) by comparison with spectral data and pure standard and due to the specific [M − H]^−^ at *m/z* 593 and a fragment at 285, corresponding to the loss of the luteolin aglycone. Interestingly, other dicaffeoyl-succinoyl quinic acid derivatives were found: one is an isomer of dicaffeoyl-succinoyl quinic acid (peak 14) and was attributed to 1,5-dicaffeoyl-4-succinoyl quinic acid (14.77 ± 0.17 mg/g dr) [42,43] and the other was attributed to a dicaffeoyl-disuccinoylquinic acid (peak 15—5.49 ± 0.10 mg/g dr) [42,43].

Several isomers of monocaffeoyl quinic acids and dicaffeoyl quinic acids were found, accounting for a total of 35.14 ± 0.18 and 25.28 ± 0.11 mg/g dr, respectively. Chlorogenic acid (5-caffeoylquinic acid, peak 5) accounted for ca. 90% of monocaffeoyl quinic acids with an amount of 31.33 ± 0.16 mg/g dr. Among flavonoids, other luteolin derivatives stand out (6.31 ± 0.02 mg/g dr). Hydroxybenzoic acids were detected in lower quantity, and the most representative were protocatechuic acid hexoside and syringic acid hexoside (0.36 ± 0.01 and 0.42 ± 0.01 mg/g dr, respectively).

Pandino et al. [44] found in leaves several mono-caffeoyl quinic acids luteolin derivatives such as luteolin rutinoside, confirming them as the most representative compounds. Interestingly, Pandino et al. [44] found monosuccinyl-dicaffeoyl quinic acids, in contrast to the dicaffeoyl-succinoyl quinic acid we found. Regarding dicaffeoyl-succinoyl quinic acids and dicaffeoyl-disuccinoyl quinic acid, they were previously found in wild cardoon leaves [42]. They were also found in other parts of the plant *C. cardunculus* [11], such as var. *altilis* stalks [43] and var. *scolymus* edible sprouts [11,43]. Notably, Pinelli et al. [42] analysed the hydroalcoholic leaf extract of wild cardoon, pointing out that the highest average value among the different classes of compounds belongs to dicaffeoyl-succinoyl quinic acids (32.5%), confirming 1,5-dicaffeoyl-3-succinoyl quinic acid as the most abundant (66.25 ± 8.79 µmol/d wt) followed by 1,5-dicaffeoyl-4-succinoyl quinic acid and 1,5-dicaffeoyl-3,4-disuccinoyl quinic acid (16.39 ± 0.36 and 6.51 ± 3.28 µmol/d wt, respectively), confirming the trend highlighted in our study. Pagliari et al. [45] confirmed hydroxycinnamic acids as the most representative classes of compounds; otherwise, among flavonoids, luteolin 7-*O*-glucoside and luteolin 7-*O*-rutinoside were the most abundant (11.99 ± 0.25 and 5.11 ± 0.16 µg/mg EXT in ethanolic extracts), in contrast with luteolin 7-*O*-rutinoside as the most abundant flavonoid in our study (31.56 ± 0.38 mg/g dr).

### 3.2. Docking Results

Molecular docking studies were conducted to evaluate the binding affinity between target phenolic compounds detected in the hydroalcoholic extract of wild cardoon and collagenase. The crystallographic structures used were human collagenase [46] with code: 1CGL and *Clostridium histolyticum* collagenase H [47] with code: 7ZOC. The co-crystallised ligands present in both structures were redocked as positive controls. The results showed good agreement between the predicted and experimental poses, with an all-atom RMSD of 1.5 Å for 1CGL and 1.3 Å for 7ZOC, confirming the reliability of the docking protocol used. The RMSD values were calculated considering all atoms of the ligand. Additionally, epigallocatechin gallate (EGCG), a compound known in the literature as a collagenase inhibitor, was also used as a further positive control to support the validity of the method. The selected compounds were the two dicaffeoylsuccinoylquinic acids (1,5-dicaffeoyl-3-succinoylquinic acid and 1,5-dicaffeoyl-4-succinoylquinic acid), luteolin 7-*O*-rutinoside, and chlorogenic acid that accounted for 74% of the phenolic compound dosed in the extract. All results of docking are shown in Table 2.

For human collagenase, the co-crystallised ligand with chlorogenic acid achieved the best XP GScore (−8.1 kcal/mol), followed by luteolin 7-*O*-rutinoside (−7.1 kcal/mol), 1,5-dicaffeoyl-3-succinoylquinic acid (−6.6 kcal/mol), 1,5-dicaffeoyl-4 -succinoylquinic acid (−5.2 kcal/mol), and EGCG (−2.9 kcal/mol). For *C. histolyticum* collagenase, 1,5-dicaffeoyl-4-succinoyl quinic acid showed the best XP GScore (−12.1 kcal/mol), followed by luteolin 7-*O*-rutinoside (−11.7 kcal/mol), and chlorogenic acid (−9.2 kcal/mol). The co-crystallised ligand and EGCG both scored –6.1 kcal/mol, while 1,5-dicaffeoyl-3-succinoylquinic acid had the lowest binding score (–3.4 kcal/mol).

**Figure 3:**
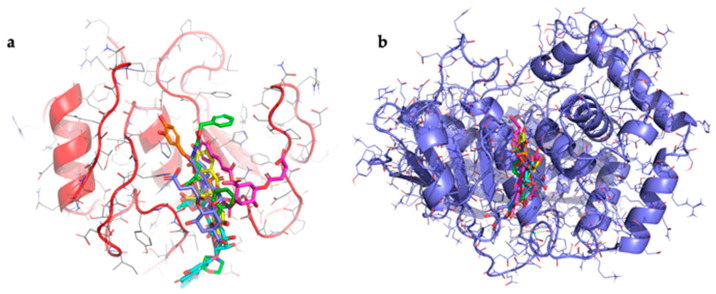
Binding mode of all compounds in collagenase: (**a**) representation of the binding mode of all compounds in human collagenase. In green: ligand 1CGL, in magenta: 1,5-dicaffeoyl-3-succinoylquinic acid, in purple: 1,5-dicaffeoyl-4-succinoylquinic acid, in yellow: epigallocatechin gallate, in cyan: luteolin 7-*O*-rutinoside, in orange: chlorogenic acid. (**b**) Representation of the binding mode of all compounds in collagenase from *Clostridium histolyticum*. In green: ligand 7ZOC, in magenta: 1,5-dicaffeoyl-3-succinoylquinic acid, in purple: 1,5-dicaffeoyl-4-succinoylquinic acid, in yellow: epigallocatechin gallate, in cyan: luteolin 7-*O*-rutinoside, in orange: chlorogenic acid.

All compounds bind on the same binding site for both collagenases used (Figure 3).

Table 2 shows that there is a high binding affinity between three of the compounds identified in the extract and *C. histolyticum* collagenase, the same one used for the experiments. These values are higher than that of the standard inhibitor. The presence of these compounds in the wild cardoon extract may help explain its better IC_50_ value compared to that of the standard ECG inhibitor in experiments with *C. histolyticum* collagenase. The data relating to the interaction between the compounds and human collagenase show that all the compounds exhibit values higher than that of EGCG. The data obtained for luteolin-7-*O*-rutinoside and chlorogenic acid agree with previously published results [48,49]. Taken together, the results help to explain the inhibitory activity of the wild cardoon extract, which contains a high quantity of these compounds.

### 3.3. Preparation of the Complex

**Figure 4:**
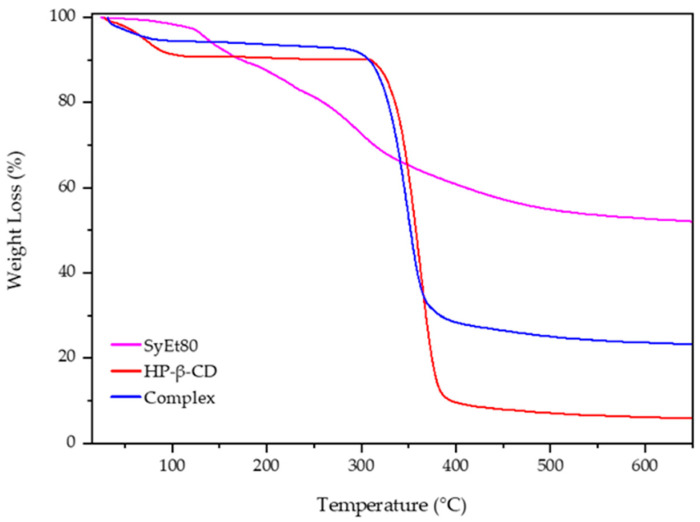
TGA thermograms of the extract/HP-β-CD complex in comparison with the free components.

The ability of CDs to act as penetration enhancers for synthetic drugs or natural molecules was now recognised [50]. Starting from this point of view, we prepared the complex between the extract and HP-β-CD to evaluate the influence of complexation on in vitro and in vivo activity of the cardoon extracts. HP-β-CD is a modified CD approved by the FDA for parenteral administration. Currently, it is present in various marketed formulations for oral, intravenous, and ophthalmic administration [51]. The solid complex was prepared by lyophilisation and analysed by thermogravimetric analysis to evaluate the influence of complexation on thermal stability of the extract. Figure 4 reports the obtained scans. The degradation of the free extract proceeds in different steps due to the complexity of its composition and the different thermal stability of the extract’s components. The first portion of the thermogram, below 130 °C, showed a very low weight loss (about 4%) due to the dehydration of the sample; then, the successive steps starting from 130 °C and finishing at about 540 °C showed the weight loss attributable to the degradation of the organic compounds and the oligosaccharides present into the extract [52], leaving a residue of about 40%, evidencing the low thermal stability of the components [53].

Free CD showed in the first portion of the curve (from 25 to 100 °C) a weight loss of about 20% (*w*/*w*) due to the evaporation of absorbed water on the macrocycle surface or present into the HP-β-CD cavity as crystallisation water [53]. After that, CD was quickly degraded (8 min) within a narrow temperature range, from 320 to 380 °C, in which a weight loss of 80% was observed due to the breakdown of the CD ring structure and a formation of 10% residue. The last stage, up to 380 °C, is characterised by the slow thermal degradation of the residue. A different trend with respect to the free extract was observed for the complex. First of all, no degradation of different compounds was observed, and the main thermal degradation of the complex proceeded in one step from 300 to 370 °C, leaving a residue of about 30%. This is the result of the complexation and improved thermal stability of the extract. Furthermore, the initial weight loss, at a temperature lower than 75 °C, is of about 10%. This different trend with respect to the free macrocycle could evidence the absence of crystallisation water; thus, the weight loss was exclusively due to the superficial water evaporation. It is conceivable that some components of the extract were included within the cavity, surprising the water molecules allocated here [54]. These results evidenced a positive influence of HP-β-CD on the thermal stability of the extract, confirming its complexation.

### 3.4. Collagenase Activity

In this study we investigated the potential of wild cardoon (SyEt80) extract to inhibit collagenase, considering the relationship between angiogenesis and collagenase activity. The IC_50_ value obtained from the free extract and in combination with hydroxypropyl-β-cyclodextrin (HP-β-CD, 0.5% *w*/*v*) (Table 3) was compared to those of the standard inhibitor to assess the extract’s inhibitory strength.

The IC_50_ value calculated for the SyEt80 extract was found to be 148 µg/mL, comparable with the standard inhibitor EGCG which has an IC_50_ value of 121 µg/mL. A more significant result was achieved with the complexed extract SyEt80/HP-β-CD, which enhanced its inhibitory activity against collagenase by 6-fold compared to the extract alone and 4.5 times more than the single molecule of EGCG. These findings demonstrate that complexation with cyclodextrin significantly enhances the extract’s inhibitory activity, most likely due to improved solubility and stability of its key phenolic compounds.

### 3.5. Chick Chorioallantoic Membrane (CAM) Assay

**Figure 5:**
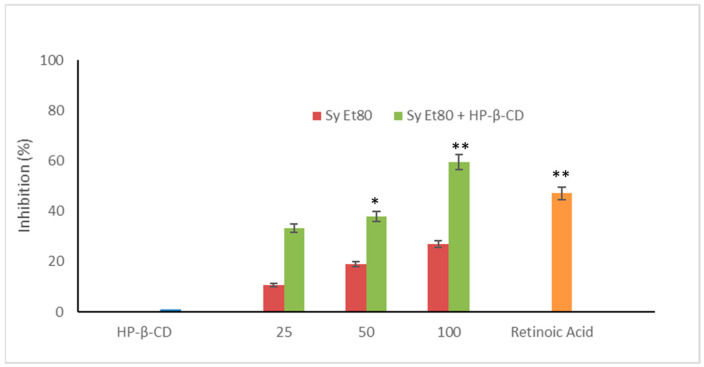
Antiangiogenic activity of SyEt80 and of SyEt80 complexed with HP-β-CD (0.5%, *w*/*v*) in the CAM assay. Results are expressed as % inhibition vs. negative control. Retinoic acid (1 µg/egg) = positive control. Mean ± SD (*n* = 10). * *p* < 0.05; ** *p* < 0.01 vs. control: Student’s *t*-test.

The antiangiogenic activity of the hydroalcoholic extract from wild cardoon leaves (SyEt80) was investigated using the chick chorioallantoic membrane (CAM) assay, both as a free extract and in combination with HP-β-CD (0.5% *w*/*v*). As shown in Figure 5, treatment with the free extract induced a dose-dependent inhibition of neovascularisation, with inhibition values of 12.20%, 18.91%, and 27.02% at doses of 25, 50, and 100 µg/egg, respectively.

**Figure 6:**
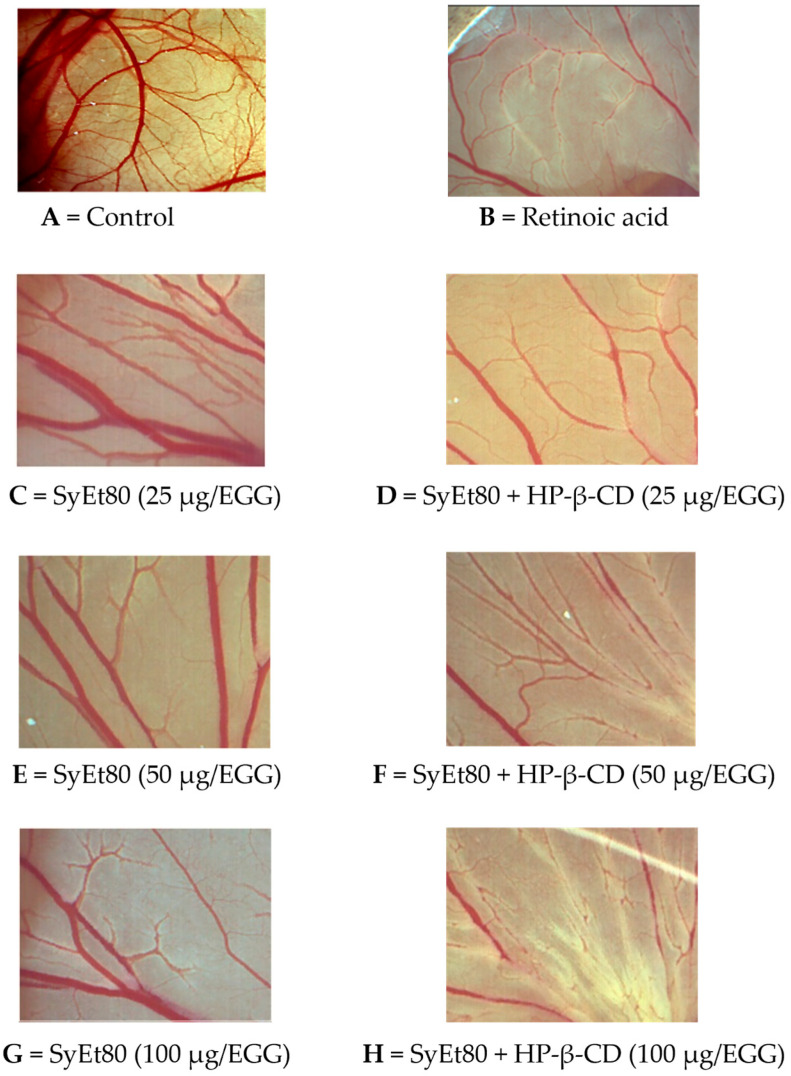
Representative images of the chick chorioallantoic membrane (CAM) assay showing the effects of SyEt80 extract and its complex with HP-β-CD on angiogenesis at different doses. (**A**) Control; (**B**) retinoic acid (positive control); (**C**–**E**) SyEt80 and (**F**–**H**) SyEt80 complexed with HP-β-CD at 25, 50, and 100 µg/egg, respectively. The images illustrate a dose-dependent antiangiogenic effect of the extract, which is enhanced by HP-β-CD complexation (scale bar = 1000 μm).

The inclusion complex SyEt80/HP-β-CD demonstrated significantly higher antiangiogenic activity, reaching inhibition percentages of 33.33%, 37.89%, and 59.45% at the same concentrations (Figure 5). Retinoic acid was used as the positive control (1 μg/egg, 44.55% inhibition). These results confirm the superior efficacy of the cyclodextrin-complexed formulation, which showed a statistically significant increase in antiangiogenic activity compared to the free extract. The improved efficacy observed in the complexed formulation supports that cyclodextrin-based delivery enhances the solubility, chemical stability, and bioavailability of the active constituents, thereby potentiating their biological effect. Representative photomicrographs of the CAMs are reported in Figure 6. As can be seen, in the control CAM there is a thick vascular network with large vessels (Figure 6A). Conversely, the retinoic acid (Figure 6B) and SyEt80/HP-β-CD (Figure 6D,F,H) treatments produced a less-dense vascular network due to the strong inhibitory effects on capillary formation.

These findings are particularly significant as they demonstrate not only the intrinsic antiangiogenic potential of wild cardoon leaf constituents but also the pivotal role of formulation strategies in modulating biological effects. The dose-dependent inhibition observed with the free extract confirms the presence of active phytochemicals able to interfere with neovascular processes. However, the marked enhancement observed with the HP-β-CD inclusion complex suggests that a considerable fraction of these bioactive compounds is otherwise limited by poor solubility or instability in aqueous environments. This aligns with literature data indicating that polyphenols such as dicaffeoylquinic acids and luteolin derivatives, abundant in the extract, benefit substantially from cyclodextrin encapsulation in terms of bioavailability and biological performance [55]. From a clinical perspective, this formulation approach not only improves the therapeutic applicability but also offers a rational strategy for the valorisation of agricultural by-products through the development of innovative, plant-based antiangiogenic agents.

### 3.6. In Vivo Antiangiogenic Potential on Zebrafish Model

**Figure 7:**
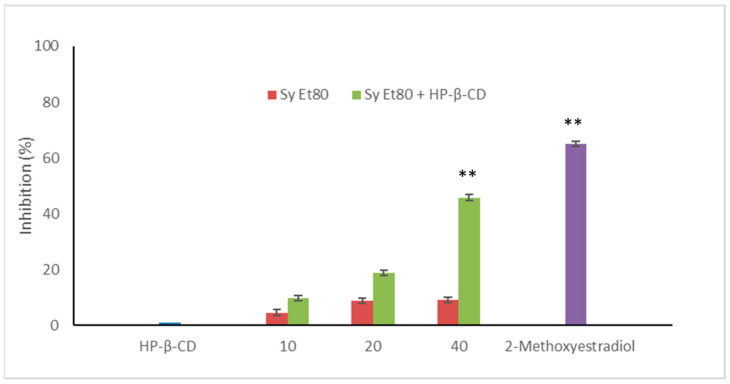
Antiangiogenic activity of SyEt80 (10, 20, and 40 μg/embryo) free and complexed with HP-β-CD (0.5%, *w*/*v*) in zebrafish embryos. Results are expressed as % inhibition vs. negative control. 2-methoxyestradiol (2 µM) = positive control. Mean ± SD (*n* = 10). ** *p* < 0.01 vs. negative control: Student’s *t*-test.

The antiangiogenic potential of the wild cardoon leaf extracts (SyEt80) and the inclusion complex with HP-β-CD (0.5% *w*/*v*) were further assessed in vivo using the zebrafish (*Danio rerio*) embryo model by measuring endogenous alkaline phosphatase (EAP) activity as a biochemical marker of vascular development. Results are reported in Figure 7.

Treatment with the free extract resulted in modest inhibitory activity, with values of 4.54%, 8.77%, and 8.03% at concentrations of 10, 20, and 40 µg/embryo, respectively. In contrast, embryos treated with the SyEt80/HP-β-CD complex exhibited a clear dose-dependent inhibition of EAP activity, with values of 9.80%, 18.15%, and 45.75% at the same concentrations. 2-Methoxyestradiol was used as a positive control (2 µM, 63.00% inhibition).

These results demonstrate that complexation with HP-β-CD markedly enhances the extract’s antiangiogenic efficacy in the zebrafish model, likely due to the improved delivery and bioavailability of its bioactive constituents. The significant increase in EAP inhibition observed with the HP-β-CD complex—especially at the highest dose—highlights how formulation strategies can profoundly influence in vivo pharmacodynamics. Considering the modest effect shown by the unformulated extract, it is reasonable to assume that the limited solubility or rapid degradation of polyphenolic compounds in aqueous solutions may compromise their biological activity in vivo. The improved performance of the inclusion complex not only confirms the functional relevance of these phenolic constituents but also underscores the value of delivery systems in addressing the solubility and stability limitations of plant-derived bioactive constituents. This evidence supports a rational, formulation-centred approach to the development of novel antiangiogenic agents from agro-industrial by-products.

Importantly, during zebrafish experiments, no signs of toxicity—such as pericardial oedema, developmental delays, or morphological malformations—were observed with either the free extract (SyEt80) or the HP-β-CD inclusion complex at the tested concentrations (10–40 μg/embryo). Embryos exhibited normal motility and developmental progression up to 72 hpf. This suggests that the antiangiogenic effects of the SyEt80/HP-β-CD complex occur without compromising embryo development. This toxicity profile supports the selection of the current dose range and reinforces the potential of the extract for future applications in the nutraceutical or functional food domain, where safety is a critical parameter.

Taken together, the findings from the CAM and zebrafish in vivo assays represent a clear demonstration of the antiangiogenic potential of wild cardoon leaf extract. The marked inhibition of neovascularisation in the CAM model and the significant suppression of vascular development biomarkers in zebrafish embryos both support a consistent biologically relevant effect. Notably, the improved efficacy of the HP-β-CD inclusion complex across both models highlights the importance of formulation strategies in maximising the therapeutic potential of plant-derived phytocomplexes.

The strength and reproducibility of these in vivo results provide evidence that the bioactive phenolic constituents of wild cardoon—particularly dicaffeoyl-succinoyl quinic acids and luteolin derivatives—are capable of modulating angiogenic mechanisms in whole-organism contexts. These findings support the potential of the extract as a promising candidate for further development in the prevention or co-treatment of angiogenesis-driven disorders. Furthermore, the consistency of results across two independent in vivo models reinforces the therapeutic relevance of the observed effects and sets the groundwork for future clinical validation.

The antiangiogenic effects observed in both the CAM and zebrafish models for SyEt80 and its inclusion complex with HP-β-CD may be mechanistically linked, at least in part, to the significant inhibition of collagenase activity. Matrix metalloproteinases (MMPs), particularly those with collagenolytic activity, are crucial mediators of the extracellular matrix (ECM) remodelling required for endothelial cell migration and vessel formation. This process represents a key step in the angiogenic cascade and is extensively documented in both physiological and pathological settings [14,15]. In our study, collagenase from *C. histolyticum* was employed as a reference enzyme due to its broad collagen-degrading activity and widespread use in preliminary screening assays for collagenase inhibitors, although it differs structurally from human matrix metalloproteinases.

In this context, results clearly demonstrate that SyEt80 and its HP-β-CD complex interfere with collagenase activity. This interpretation is further supported by molecular docking analyses performed on both bacterial and human collagenase structures, which revealed that the major phenolic constituents of the extract bind to the same binding site for both collagenases used. This mechanistic hypothesis is consistent with prior studies showing that angiogenesis is significantly reduced when collagenase-mediated ECM degradation is suppressed [14], and is further supported by comprehensive reviews on the multifunctional role of MMPs in neovascularization.

However, collagenase inhibition is unlikely to be the sole explanation for the observed antiangiogenic effects. Polyphenolic compounds, which are abundantly present in SyEt80, are known to modulate angiogenesis through a range of multiple and interconnected mechanisms—such as the inhibition of VEGF signalling, antioxidant activity, and the downregulation of inflammatory mediators [12,15]. Therefore, it is plausible that the anti-angiogenic activity of the extract is the result of a multi-targeted mechanism, in which collagenase inhibition represents a relevant, but not exclusive, contributor.

## 4. Conclusions

The in vitro and in vivo findings of this study provide evidence of the anti-angiogenic potential of *Cynara cardunculus* L. subsp. *cardunculus* (wild cardoon) leaf extracts. Notably, the inclusion complex of the extract with hydroxypropyl-β-cyclodextrin markedly enhanced the inhibition of collagenase activity and angiogenic process. The improved activity of the complexed extract is likely due to the increased solubility and stability of key phenolic constituents.

The consistent inhibition observed across both systems highlights the therapeutic relevance of this phytocomplex and the importance of effective formulation strategies. These results suggest that wild cardoon leaf by-products, often considered agricultural wastes, may represent a sustainable and effective source of collagenase inhibitors and antiangiogenic agents. These results could be used for future applications in the management of collagenase and angiogenesis-related disorders. Furthermore, the valorisation of wild cardoon leaf by-products offers promising opportunities beyond pharmaceutical applications. Due to their high content of health-promoting phenolic compounds and favourable profile, these by-products are also suitable for incorporation into functional food products or nutraceutical formulations designed to support vascular health. This dual potential not only broadens their scope of application but also aligns with the principles of circular bioeconomy, providing a sustainable model to transform agro-industrial wastes into high-value bioactive ingredients with significant health benefits.

## Figures and Tables

**Table 1:** Concentration of targeted phenolic compounds detected in wild cardoon extract (mg/g dry extract, mean ± SD; *n* = 3).

Compound	Number Figure 2	Wild Cardoon Extract (mg/g dr)
		Mean ± SD
**Total hydroxycinnamic acids**		**119.33 ± 0.77**
Mono caffeoylquinic acids ^a^	2, 3, 5, 6	35.14 ± 0.18
Dicaffeoylquinic acid ^b^	8, 9, 10, 12	25.28 ± 0.11
Dicaffeoyl-succinoylquinic acid I ^b^	13	38.64 ± 0.21
Dicaffeoyl-succinoylquinic acid II ^b^	14	14.77 ± 0.17
Dicaffeoyl-disuccinoylquinic acid	15	5.49 ± 0.10
**Total flavonols**		**37.87 ± 0.40**
Luteolin 7-*O*-rutinoside	7	31.56 ± 0.38
Other luteolin derivatives ^c^	11, 16	6.31 ± 0.02
**Total hydroxybenzoic acids**		**0.78 ± 0.02**
Protocatechuic acid hexoside ^d^	1	0.36 ± 0.01
Syringic acid hexoside ^e^	4	0.42 ± 0.01
**Total polyphenols**		**157.98 ± 1.19**

^a^ Dosed as chlorogenic acid equivalents; ^b^ dosed as 1,3-di-*O*-caffeoylquinic acid equivalents; ^c^ dosed as luteolin equivalents; ^d^ dosed as protocatechuic acid equivalents; ^e^ dosed as syringic acid equivalents.

**Table 2:** Docking results for the interactions of compounds with the human collagenase (1CGL code) and *Clostridium histolyticum* collagenase (7ZOC code). The results are all expressed in kcal/mol.

Compound	XP-Score 1CGL	XP-Score 7ZOC
Epigallocatechin gallate (EGCG)	−2.9	−6.1
1,5-dicaffeoyl-3-succinoylquinic acid	−6.6	−3.4
1,5-dicaffeoyl-4-succinoyl quinic acid	−5.2	−12.1
Luteolin 7-*O*-rutinoside	−7.1	−11.7
Chlorogenic acid	−8.1	−9.2
Ligand 1CGL	−8.1	
Ligand 7ZOC		−6.1

**Table 3:** Inhibitory effects (IC_50_) of SyEt80 extract and SyEt80/HP-β-CD on collagenase enzymatic activity. Epigallocatechin gallate is reported as standard inhibitor. All data represent the mean ± SD of three independent experiments.

Sample	IC_50_ (µg/mL)
SyEt80 extract	148.8 ± 0.5
SyEt80/HP-β-CD	26.8 ± 2.0
Epigallocatechin gallate	120.8 ± 6.2

## Data Availability

The original contributions presented in the study are included in the article, further inquiries can be directed to the corresponding authors.
